# Different Methods of Teaching and Learning Dental Morphology

**DOI:** 10.3390/dj12040114

**Published:** 2024-04-18

**Authors:** Helene Lindén Overskott, Caroline Elisabet Markholm, Amer Sehic, Qalbi Khan

**Affiliations:** Institute of Oral Biology, Faculty of Dentistry, University of Oslo, Blindern, P.O. Box 1052, 0316 Oslo, Norway; heleneld@student.odont.uio.no (H.L.O.); carolem@student.odont.uio.no (C.E.M.); qalbi.khan@odont.uio.no (Q.K.)

**Keywords:** dental anatomy, dental education, digital learning, e-learning, tooth identification

## Abstract

Dental anatomy education is traditionally structured into theoretical and practical modules to foster both cognitive and psychomotor development. The theoretical module typically involves didactic lectures where educators elucidate dental structures using visual aids. In contrast, practical modules utilize three-dimensional illustrations, extracted and plastic teeth, and tooth carving exercises on wax or soap blocks, chosen for their cost, ease of handling, and fidelity in replication. However, the efficacy of these traditional methods is increasingly questioned. The criticism in this concern is that oversized carving materials may distort students’ understanding of anatomical proportions, potentially affecting the development of necessary skills for clinical practice. Lecture-driven instruction, on the other hand, is also criticized for its limitations in fostering interactive learning, resulting in a gap between pre-clinical instruction and practical patient care. In this study, we review the various educational strategies that have emerged to enhance traditional dental anatomy pedagogy by describing the effectiveness of conventional didactic lectures, wax carving exercises, the use of real and artificial teeth, the flipped classroom model, and e-learning tools. Our review aims to assess each method’s contribution to improving clinical applicability and educational outcomes in dental anatomy, with a focus on developing pedagogical frameworks that align with contemporary educational needs and the evolving landscape of dental practice. We suggest that the optimal approach for teaching tooth morphology would be to integrate the digital benefits of the flipped classroom model with the practical, hands-on experience of using extracted human teeth. To address the challenges presented by this integration, the creation and standardization of three-dimensional tooth morphology educational tools, complemented with concise instructional videos for a flipped classroom setting, appears to be a highly effective strategy.

## 1. Introduction

The study of tooth morphology constitutes a profound element in the dental curriculum, emphasizing the necessity for dental students to develop a complete and detailed knowledge of the intricate structure of teeth [1]. A comprehensive understanding of tooth anatomy, which includes its characteristics, similarities, differences, and variations, is crucial and applicable to every aspect of the dental profession. This knowledge is essential for nearly all clinical dental procedures, including restorative dentistry, endodontics, oral surgery, orthodontics, prosthodontics, pediatric dentistry, periodontics, dental radiography, and oral pathology [2,3]. Furthermore, dental morphology holds significant relevance in fields that extend beyond direct patient care, such as forensic investigations, anthropological research, and the examination of fossilized remains in paleontological studies.

Gaining proficiency in tooth morphology can be approached through various educational methods and pedagogical approaches, such as didactic lectures [4], dental anatomy carvings [5], the study of extracted teeth [6], the use of plastic teeth [1], the examination of a variety of tooth illustrations and images [2], and different e-learning tools [4,7]. Nonetheless, all these techniques exhibit certain drawbacks and/or difficulties. Some of the limitations identified pertain to the static nature of images that cannot replicate the three-dimensional complexity of teeth, the ethical and practical issues about the use of real extracted teeth, and the potential disparity between the tactile experience provided by wax or plastic models and that of real dental tissues, along with the loss of morphological variations found in the plastic models. These constraints highlight the necessity for educational innovation in the field of dental morphology to better equip students for the demands of clinical practice.

Contemporary dental students are generally adept at using technology for learning, which indicates that adding digital learning tools to the dental curriculum could be both convenient and agreeable [7,8]. Online quizzes and tests are known to engage students and enhance learning [4], and increasingly, novel approaches containing different e-learning elements are now being used for teaching tooth morphology [9]. Advances in computerized tomography (CT) and micro-CT scanning technologies have improved our ability to visualize tooth root anatomy [10], develop dental atlases, and create three-dimensional models of the pulp chamber and teeth [11]. These tools allow many students to view materials at once and are useful in various educational settings. Combining these digital strategies with traditional teaching approaches can address different learning styles effectively. However, mixing too many varying digital tools, without clear pedagogical aims, may lead to a disorganized teaching program and cause confusion and frustration for both the students and the teaching staff.

The curriculum of dental anatomy conventionally comprises theoretical instruction and a practical component to cultivate both cognitive understanding and psychomotor abilities. Traditional pedagogical practices are prevalent in most dental institutions, utilizing lectures for theoretical elucidation where an instructor explicates the anatomical characteristics of teeth via slideshows or drawings on white/black boards. Despite the longstanding application of these conventional methods in dental anatomy education across the world, their effectiveness has come under scrutiny. A notable criticism is the lack of integration of dental anatomy courses with other patient care-related courses, resulting in insufficient emphasis on clinical applicability [5]. This traditional approach often does not effectively transition students from pre-clinical education to actual patient care settings [1]. Furthermore, the lecture-based model is constrained by unidirectional communication, limited interactivity, and suboptimal student engagement [12]. These limitations highlight the insufficiency of theoretical knowledge in acquainting students with detailed dental anatomy.

Extracted teeth are widely acknowledged as invaluable teaching aids due to the anatomical variations they exhibit, although they raise ethical and health-related concerns. Dental students typically receive instruction in tooth morphology during the initial two years of their curriculum, but its practical application occurs during the clinical years, often leading to fragmented learning experiences [4,13]. To address these challenges, the development and standardization of 3D tooth morphology teaching tools may offer a solution [14]. These tools can relieve the pressure of retaining anatomical specimens and facilitate a more comprehensive integration of tooth morphology instruction across all years of dental education. While foundational tooth morphology courses should continue in the pre-clinical years, several studies have shown and recommended encouraging ongoing skill development using standardized 3D visualization tools and periodic reassessment as students progress toward degree completion.

Although several recent reviews have aimed to consolidate various educational strategies that have been proposed or implemented to transcend traditional anatomy teaching methodologies, few have focused specifically on the teachings of tooth morphology, which requires a distinct staff, professional background, anatomical resources, and student group.

The objective of this chapter is to highlight, evaluate, and discuss the educational tools and methodologies applied in the study of tooth morphology, particularly in the context of an evolving digital educational landscape. A critical comparison of these methods is essential to understand how traditional techniques can be enhanced using digital innovations. The integration of classical approaches with modern technology-based strategies offers a composite educational model that could potentially yield more comprehensive learning outcomes. This study seeks to explore the effectiveness of different teaching modalities, both stand-alone and in synergy, to determine the optimal blend of resources that can cater to diverse learning preferences.

**Figure 1:**
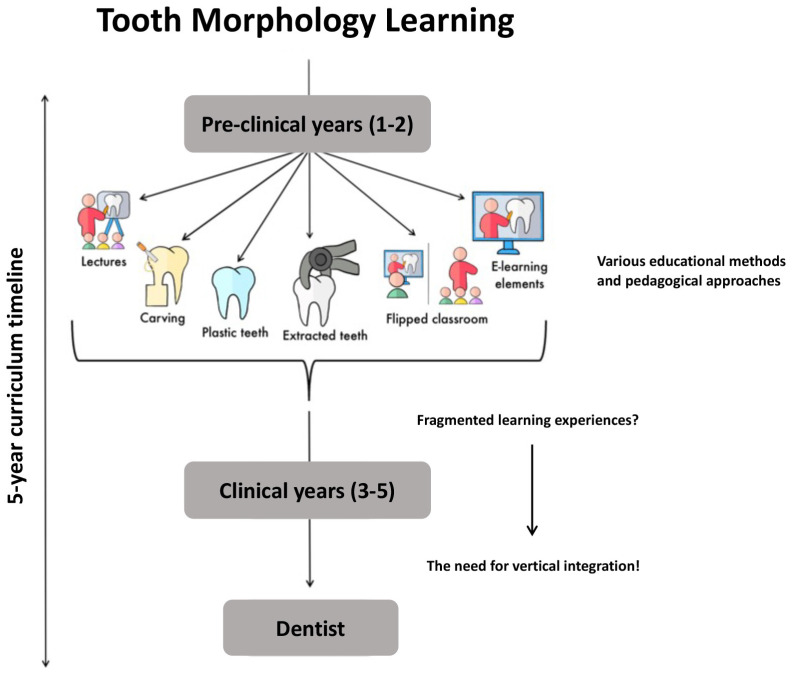
This figure delineates the study methods and evaluation points employed in the instruction of tooth morphology across a 5-year curriculum timeline. The foundational aspects of dental morphology are introduced during the initial two years (years 1 and 2), while the practical application of this knowledge occurs during the subsequent clinical years, which can lead to fragmented learning experiences. The imperative need for vertical integration becomes evident in the subsequent years (years 3–5) of the curriculum.

With a specific focus on tooth morphology teaching modalities, we evaluate the pedagogical efficacy of didactic lectures, wax carving exercises, the incorporation of plastic and extracted teeth, the flipped classroom model, and the diverse components of e-learning, with an overarching goal of enhancing the clinical relevance and educational outcomes in dental anatomy. The literature search was conducted in the National Library of Medicine database using the following search terms and combinations of keywords: “tooth, dental, anatomy, morphology, identification, teaching, learning, and education”. Additional relevant studies, found referenced in the searched literature, were also evaluated. An overview is described below and presented as a table (Figure 1, Table 1).

## 2. Overview of the Current Teaching Strategies

### 2.1. Didactic Lectures

Previous studies indicate varied perceptions among educators regarding the relative importance of lecture versus practical sessions in the teaching of tooth morphology, with the majority favoring a more condensed lecture approach complemented by practical sessions [6,9,26]. In an investigation into the pedagogical approaches to teaching tooth morphology in dental education across the United Kingdom and Ireland, the study scrutinized the quantity of lecture and practical session hours allocated annually by the faculty. The data delineated a predominant inclination toward limited lecture durations, with 81.25% of the respondents, equivalent to thirteen educators, imparting between zero to ten hours of lectures on tooth morphology per year, suggesting a consensus among a significant majority regarding the sufficiency of a concise lecture format for the subject matter [27].

Didactic lectures using digital slide presentation programs, such as PowerPoint, have been widely recognized as an effective medium for teaching dental anatomy, as confirmed by various studies [9,15]. The nature of dental anatomy, with its intricate structures, makes it particularly suited to visual representation, which PowerPoint facilitates well. These lectures often utilize multiple two-dimensional images, aiding students in conceptualizing three-dimensional structures, which is a critical aspect of understanding dental anatomy. However, the effectiveness of lectures based on presentation programs is not limited to visual learning alone. These lectures can be enriched with other teaching modalities, providing a holistic and multimodal educational approach. While presentation programs serve as a foundational tool, the integration of various methods can enhance the learning experience, catering to diverse learning preferences among students.

The shift to online education during the recent COVID-19 pandemic led to a significant global increase in the use of webinars and online lectures in dental schools [16,19]. This transition highlighted the versatility and adaptability of digital teaching methods. As educational institutions returned to traditional face-to-face teaching, the value of these recorded lectures persisted. Webinars and online lectures offer a supplementary resource for students, aiding in the revision and deepening of understanding at a student’s self-chosen pace, which is particularly beneficial for both visual and auditory learners. Research by Bacro and colleagues underscores the importance of these resources for auditory learners [17]. Their study revealed a notable correlation between students who prefer auditory learning and an improvement in their grades when they frequently accessed lecture recordings. This suggests that providing recorded lectures can be a significant aid in the learning process, especially for those who benefit from auditory repetition and reinforcement. Thus, while PowerPoint remains a cornerstone in teaching dental anatomy, the inclusion of recorded lectures and other teaching methods creates a more inclusive and effective learning environment.

Collectively, traditional lectures, most of them using PowerPoint, have for many years been universally favored by respondents for delivering lectures, as it enables the inclusion of key multimedia elements like visual cues and animations, which enhance learning [18]. Despite this, studies revealed that many educators also used a variety of additional methods alongside PowerPoint, such as physical models, videos, and more animations. These approaches, blending visual and interactive elements, are crucial for developing a deep understanding of anatomical structures [28]. Animations are effective in engaging students and enhancing their learning experience. Moreover, these blended learning strategies accommodate various learning styles and have been demonstrated to yield superior results compared with conventional lecture methods [29,30].

### 2.2. Wax Carving Pedagogy

Traditional methodologies in dental education, particularly in the realm of tooth morphology, have relied heavily on the use of various media for carving tooth models, such as wax, chalk, or soap. This technique, documented in a range of studies [8,9], serves not only as a fundamental pedagogical tool but also to intricately understand complex dental morphology. The haptic and visual engagement in tooth carving exercises fosters the development of fine motor skills and provides a concrete basis for the theoretical knowledge required for the reconstruction of tooth structures in restorative dental procedures [2,19]. A landmark study by Abu Eid and colleagues revealed that such practical sessions significantly bolstered first-year graduate dentistry students’ manual dexterity, as well as their comprehension and visualization of the teeth’s three-dimensional architecture [2].

Expanding upon this foundation, traditional carving exercises were integrated with clinical-based teaching paradigms and digital learning platforms [31]. This hybrid educational strategy aimed to elevate student engagement and knowledge retention by demonstrating the clinical relevance of tooth anatomy and acquainting students with the digital modalities increasingly employed in contemporary dental practices. The exigencies of the COVID-19 pandemic necessitated innovative educational adaptations, leading to the implementation of at-home waxing exercises and the use of 3D tooth models [16,19]. These measures were recognized for their effectiveness in imparting critical didactic content despite the constraints of remote learning. However, the student body exhibited a collective inclination toward resuming on-site educational experiences for tooth morphology modules, indicating a preference for the traditional, interactive learning environment.

The debate regarding the role of dental carving exercises within the dental curriculum persists, with some scholars advocating for their continued use, citing the enhancement of detailed morphological comprehension and the introduction of essential three-dimensional spatial concepts and manual instrumentation skills [19,20]. These competencies are not only academically beneficial but are also integral to the practical dental care of patients. However, concerns have been raised related to cost–benefit reasoning, as waxing courses, along with being time-consuming, also rely on resources such as tools and wax. Based on personal experience and the allowing climate, this hurdle may be creatively tackled using snow as carving material in outdoor courses. Even though the arctic climate in this case has its obvious geographical advantages, the idea should be properly investigated for learning outcomes, as malleable materials such as clay and mud may be reasonable costless options in other places.

In summary, the pedagogical value of wax carving in imparting comprehensive knowledge of dental morphology is a subject of ongoing debate within the academic community. Critics point out that despite the development of manual skills, wax carving may not offer an adequate return on the investment of time when considering the lack of exposure to the natural diversity found in tooth morphology. Furthermore, carving models, being uniform, fail to replicate the range of anatomical variations that students are likely to encounter in a clinical setting. Furthermore, even though this method provides the opportunity to enhance the typical morphological features of a tooth, the modeled tooth often fails to represent the size and hardness of a natural tooth. Despite these criticisms, the prevalence of wax carving as a teaching tool is substantiated by a substantial volume of the recent academic literature, indicating a sustained interest and perceived value in this educational method. The debate over its efficacy underscores a broader discussion on the adaptation of dental education to encompass a variety of learning styles and the need for innovative teaching methods that can sustain student engagement while providing a comprehensive education in tooth morphology.

### 2.3. Plastic and Extracted Human Teeth

For many years, plastic teeth have served as an important resource for teaching and assessing tooth morphology in educational settings. The comprehension of anatomical morphology and its diverse variations is imperative for the accurate identification of pathological conditions. Although plastic teeth are readily accessible and widely employed in teaching, they do not provide the diverse range of variations observed in extracted teeth [6,9]. Therefore, throughout the history of dental education, extracted teeth have been extensively utilized as a primary pedagogical tool for studying dental morphology and identifying specific tooth characteristics. The preference for this traditional method among dental students is well documented, underscoring the favored use of extracted teeth for studying the intricacies of dental morphology [2]. However, this approach is not without its challenges. The procurement of an adequate supply of hygienic, non-carious, and unworn extracted teeth that have been collected with informed consent presents a logistical and ethical obstacle [14]. Moreover, the advancements in oral healthcare and the corresponding decline in tooth extractions have exacerbated the scarcity of suitable specimens for educational use [32]. This shortfall has catalyzed a search for innovative methods to supplement traditional teaching aids [1]. In response to these challenges, dental education curricula are increasingly integrating novel teaching methods that employ contemporary technologies to facilitate the visualization and three-dimensional understanding of dental morphology [11,14,16]. These innovative approaches, including digital simulations and 3D printing, offer alternative means to convey the complex anatomical structures of teeth [8].

Despite the advent of these new educational tools, the use of extracted teeth remains a cornerstone in the learning and assessment of dental morphology. Their tangible nature provides an irreplaceable depth of learning through direct handling and observation, which is still highly valued in dental education today [33]. As such, while alternative methods are being adopted, the use of extracted teeth continues to be a preferred and indispensable component in the study and examination of tooth morphology within dental curricula. In the next section, we elaborate on the enduring significance of extracted teeth in the context of dental morphology education and learning.

### 2.4. Flipped Classroom

The flipped classroom model, alternatively known as the inverse or backward classroom, is an educational strategy situated within the domain of blended learning. It subverts the traditional educational paradigm by combining online learning with direct classroom interaction. Employing computer technology, this method reverses the conventional learning environment by delivering educational content primarily outside of the classroom. Students first engage with fundamental concepts through digital multimedia tools like concise videos and prerecorded lectures accessible online [34]. Subsequently, the classroom becomes a space for the detailed exploration and discussion of these concepts, ideally within a collaborative, group-based learning setting.

The fusion of the flipped classroom approach with online educational resources has been shown to markedly improve student engagement and support the learning process within dental anatomy programs [1,2,13,35]. Noteworthy is the initiative by the University of Illinois at Chicago, which introduced a novel, indexed literature-based approach to enrich the dental anatomy curriculum [1]. This model synergizes autonomous pre-class preparation with dynamic group discussions and practical wax-up exercises using mannequins for a realistic representation of tooth structure [1]. In parallel, Bakr and colleagues at Griffith University deployed a hybrid flipped model, providing a suite of online videos, a digital library, and electronic quizzes [35]. Abu Eid and associates also recounted the evolution of the dental anatomy course at the University of Aberdeen Dental School, which transitioned from conventional methods to a system reliant on online materials and instructor-led, self-directed workshops [2]. These changes are aligned with transformations implemented at prestigious institutions such as the Harvard School of Dental Medicine [21,22] and the University of Glasgow [23]. The overarching aims of these innovative educational redesigns are to utilize classroom time for interactive group discussions and hands-on practice, deliver core content outside of class through digital means, and facilitate a smoother transition for students from theoretical learning to clinical practice [1].

The implementation of the flipped classroom design in dental anatomy education holds considerable promise in revolutionizing the instruction of tooth morphology in dental schools. By integrating cognitive and psychomotor learning, this approach smoothens the progression to patient-centered care in a clinical setting [22,35]. The advantages of this model are manifold. It creates a student-centric learning environment, empowering learners to assume responsibility for their educational journey. Here, the instructor acts more as a facilitator in the learning process than a direct conveyor of information [2,22]. The availability of digital resources allows for students’ unfettered access to learning materials, fostering interactivity and self-directed learning, leading to active engagement in the learning process [34]. By transferring the delivery of basic content online, the model makes efficient use of in-person class time to engage students in activities that promote critical thinking and a team-based learning (TBL) approach. It also supports the incorporation of simulation labs, enriching the learning experience and ensuring coherence with broader pre-patient and clinical curricula. These activities are instrumental in honing psychomotor abilities crucial for dental practice, such as visual discernment and the dexterity required to reconstruct normal tooth anatomy and undertake clinical procedures. Additionally, the flipped classroom format provides students with the opportunity to assess their work critically and improve upon it through immediate feedback from their peers and instructors [22,34,36].

Nonetheless, this approach is not without its challenges. A primary concern is the dependence on students’ intrinsic motivation and self-regulation to adequately prepare before each classroom encounter. To address this constraint, an approach comprising the administration of low-stakes quizzes prior to class sessions could be adopted to enhance student adherence to required preparations. Concurrently, apprehensions have been voiced about the potential augmentation in students’ workload and the associated time commitment required for preparation. Hence, it is crucial to judiciously calibrate students’ workload by maintaining an equilibrium between the preparatory materials and the establishment of lucid educational objectives and anticipated learning outcomes [37,38].

### 2.5. E-Learning Elements

The adoption of e-learning, particularly interactive media, marks a significant departure from the traditional linear lecture format, which is acknowledged for its limited interactivity. In contemporary dental education, the current cohort of dental students belongs to a distinct generational paradigm characterized by a profound affinity for technology. This generational shift is underscored by the ubiquitous integration of technology into their lives, rendering it a fundamental assumption in their learning journey. These learners exhibit heightened visual literacy and a seamless ability to navigate between physical reality and virtual realms with remarkable ease. In contrast to conventional linear thought processes, the hallmark of this generation lies in their aptitude for amalgamating information from diverse sources [7]. Researchers scrutinizing this demographic assert that they possess an innate proficiency in visual communication, demonstrating an exceptional capacity to swiftly shift their focus from one task to another. These students are adept at swift responses and anticipate prompt reciprocation. Notably, scholarly investigations indicate their inclination toward shorter textual content and a proclivity for immersive, image-rich environments over conventional text-based platforms [24]. They are often characterized as computer-savvy individuals fostering an expectation for multimedia, experiential, and interactive pedagogical approaches. In their educational experience, these students demand interactivity as a pivotal instructional component, and technology empowers the provision of content and interaction at any time and place. Consequently, computer-based instruction surpasses traditional lectures in fostering interactivity, a quality that resonates profoundly with this emerging student generation.

Recent research, such as the study conducted by Obrez et al., underscores that, when appropriately scaffolded, students exhibit a greater capacity for autonomous learning through e-learning in contrast to conventional lecture-based courses [1]. E-learning offers students multiple avenues for acquiring, assimilating, and revisiting information. Numerous studies spanning various domains of higher education have consistently demonstrated that students’ performance in e-learning environments is on par with what is typically achieved through traditional classroom lectures, with no compromise in learning outcomes. In particular, Bogacki et al. found that e-learning strategies employing computer-animated graphics for teaching human dental morphology yielded statistically equivalent results compared with traditional lecture methods [4].

Further corroborating these findings, a study revealed that an independent, interactive e-learning module dedicated to dental morphology substantially enhanced foundational knowledge acquisition, leading to statistically significant improvements in didactic examination performance [7]. The interactive nature of the content effectively engaged the new generation of dental students, who recognized the module as a valuable learning resource. However, while preferred over traditional classroom settings, e-learning was not considered a complete replacement. Instead, the integration of e-learning into the curriculum was deemed more effective when complemented by some in-person activities or seminars to fulfill students’ desire for faculty interaction. The efficacy of the interactive e-learning materials was contingent on their compulsory inclusion, as opposed to being merely recommended [7]. These findings offer compelling support for the affirmative impact of independent, interactive e-learning modules on learning outcomes, consistent with results from previous research [39]. One plausible explanation for this phenomenon may lie in the inclusion of self-paced, interactive quizzes embedded within lecture videos, which likely contributed to a deeper understanding of the material, aligning with prior research findings [25].

### 2.6. Tooth Identification Puzzle Using Extracted Human Teeth

At the Institute of Oral Biology within the Faculty of Dentistry at the University of Oslo, our innovative pedagogical method known as the “tooth identification puzzle” approach has been longstanding in the curriculum for teaching tooth morphology [6]. This method emphasizes the tangible engagement with real teeth, recognizing the integral role of tactile and visual interactions in the comprehensive understanding of dental anatomy. A recent evaluation of this approach involved a post-course examination designed to assess the proficiency gained in tooth identification, necessitating an intimate knowledge of tooth morphology. The course was structured to provide an in-depth understanding of the subject within a condensed timeframe and was anticipated to be both stimulating and enjoyable for the participants. Initially, students were introduced to the core concepts of the subject through two or three 45 min lectures. Following this theoretical foundation, an 11 to 12 h practical segment was conducted, wherein students engaged in the hands-on identification of teeth. The practical component utilized sets of extracted teeth, which included the complete series of 32 permanent teeth and 8 deciduous molars. The task for the students involved correctly identifying and placing each tooth in a schematic dentition diagram, employing the FDI World Dental Federation notation system [6].

The adoption of a “tooth identification puzzle” pedagogy in teaching tooth morphology presents a myriad of advantages for both educators and students, as discerned through our academic experience. This approach is not only cost-efficient but also requires a manageable duration of approximately 14–16 h for course completion. Student feedback, garnered from both formal and informal post-course evaluations, has been overwhelmingly positive. The gamified aspect of the curriculum significantly augments the learning process, making it an enjoyable experience while simultaneously facilitating substantial skill advancement among the students. The initial stages of the course are characterized by a deliberate pace as students acclimate to the foundational principles that govern tooth architecture, become conversant with the specialized nomenclature, and discern the subtle distinctions and similarities across different teeth. A transformative moment typically arises at the course’s midpoint, marked by what students describe as an “aha” moment when the conceptual pieces start to coalesce, leading to an acceleration in their learning trajectory. This phase of enlightenment is evidenced by an observable escalation in the accurate placement of teeth and a decrease in the time required to complete this task, which is indicative of their progressing proficiency.

The course’s efficacy stems from an immersive learning environment that incorporates direct observation, the physical manipulation of teeth models, collaborative discussions with peers and instructors, and extensive consultation with compendium text and illustrative figures. This rich sensory engagement and dynamic interaction serve as potent catalysts for an effective educational experience [40,41]. The course further acquaints students with the inherent variability in tooth morphology, providing a hands-on understanding of diverse dental structures through the examination of various tooth sets. This experiential learning is instrumental in preparing students for real-world dental challenges. The collaborative atmosphere of the course also lays the foundation for a positive and interactive relationship between students and faculty, which is pivotal for a nurturing educational milieu, thereby promoting not just academic learning but also student well-being.

Our observations have also highlighted common challenges encountered in the learning process. One of the most frequent difficulties faced by students is the accurate determination of a tooth’s orientation, especially for symmetric teeth like the central mandibular incisor and the second maxillary premolar. However, this issue also persists for teeth that are notably asymmetric, such as the first maxillary premolar and the first and second temporary molars, indicating that the challenge lies more in the spatial orientation rather than in the recognition of asymmetry traits. Additionally, our experience points to certain persistent issues in the tooth identification process. Firstly, students show variability in their apprehension and progression through the course material; however, they universally reach a satisfactory level of understanding. Secondly, the angle of observation is critical when assessing morphological features—certain perspectives, such as from the incisal or occlusal view, are imperative for accurate assessment. Thirdly, identifying the facial aspect of a tooth, particularly for molars, is crucial for correct side determination. A noteworthy teaching point is the convergence patterns of a tooth’s surfaces, which are essential for orientation. Lastly, the tactile exploration of subtle dental features, such as root furrows, plays a significant role in comprehensive learning.

Though we have not conducted systematic evaluations of the retention of tooth morphology knowledge in the latter stages of the curriculum, preliminary assessments, such as identification tests included in end-of-semester examinations, have yielded satisfactory results. Furthermore, the performance of students in clinical settings, coupled with feedback from their instructors, suggests that the knowledge gained from the course is effectively applied in practice. Future studies could be beneficial to quantitatively assess knowledge retention and its application in clinical practice to refine educational strategies further.

## 3. Conclusions and Future Perspectives

Proficiency in tooth anatomy holds paramount importance across various dental disciplines. Tooth morphology necessitates the visualization and accurate understanding of dental features and their dynamic interactions. It constitutes a pivotal component of the dental curriculum, although students may not immediately recognize its significance, and they may not retain all the detailed information throughout their clinical years and future careers. Several studies have underscored that dental schools have adopted diverse approaches to teaching tooth morphology, as described in the literature review. The rapid evolution and availability of teaching supplements have facilitated the transition from lecture-based or extracted teeth-focused curricula to more versatile programs accommodating various learning styles and offering a high degree of flexibility, particularly as more intricate programs are developed.

Didactic lectures still hold a firm position in the teaching of tooth morphology, as programs like PowerPoint enable the blending of videos and animations. However, to develop a deep understanding of anatomical details, a practical course such as dental anatomy carvings or the use of plastic teeth is deemed essential. E-learning tools, independent and interactive, may serve as a valuable supplement. Extracted teeth remain widely recognized as invaluable educational tools due to their ability to showcase various anatomical variations, despite concerns related to ethics and health and fragmented learning experiences. While foundational tooth morphology courses should continue in the pre-clinical years, it has been underscored in multiple studies that encouraging the ongoing development of skills through the utilization of standardized 3D visualization tools, brief videos, as in a flipped classroom, that explain key concepts and provide periodic reassessment as students’ progress toward degree completion is crucial.

In conclusion, we opine that the most suitable program for tooth morphology teaching is one that successfully merges the digital aspects of the flipped classroom into course-based learning that applies the tooth identification puzzle method using extracted human teeth. To tackle the challenges related to this, the development and standardization of 3D tooth morphology teaching tools, along with brief videos to suit the flipped classroom strategy, emerge as a promising solution. These tools not only alleviate the pressure of preserving anatomical specimens but also facilitate a more comprehensive integration of tooth morphology instruction throughout the entire dental education program.

## Figures and Tables

**Table 1:** Teaching methods used in tooth morphology education, including their main advantages and drawbacks.

Teaching Method	Main Advantages	Main Drawbacks	References
**Didactic lectures**	- Important overview - Visual representation - Multimodal educational approach	- Two-dimensional images - Reduces interactive learning - Decreases student engagement	Lone et al. [9] Shigli et al. [15] Lone et al. [16] Bacro et al. [17] Moreno et al. [18]
**Wax carving pedagogy**	- Instrumentation skills - Three-dimensional architecture - Provides a basis for the reconstruction	- High investment of time - Lack of natural diversity of teeth - Less anatomical variation - Incorrect anatomical proportions	Abu Eid et al. [2] Maggio et al. [7] Mitov et al. [8] Goodacre et al. [19] Lone et al. [16] Conte et al. [20]
**Plastic teeth**	- Easily accessible - The size may be like the size of real extracted teeth	- Less anatomical variation - Do not represent real dental tissues	Risnes et al. [6] Lone et al. [9]
**Extracted human teeth**	- Real morphology and size - Direct handling and observation - Anatomical variations - Enhances student engagement	- Ethical and practical issues - Hygiene concerns - Hard to obtain teeth without caries or fillings	Obrez et al. [1] Risnes et al. [6] Mitov et al. [8] Lone et al. [9] Lone et al. [16]
**Flipped classroom**	- Combines online learning with classroom interaction - Educational content outside the classroom - Enhances student engagement - Transition to clinical practice	- Requires student self-discipline - Heavy reliance on student self-motivation - Increased workload for students	Obrez et al. [1] Bakr et al. [3] Chutinan et al. [21] Park et al. [22] Crothers et al. [23]
**E-learning elements**	- Greater capacity for autonomous learning - Multiple avenues for acquiring and revisiting information - Engages the new generation of dental students	- Cannot be considered a complete replacement for “real” interactions with teeth - Requires student self-discipline - Heavy reliance on student self-motivation	Obrez et al. [1] Bogacki et al. [4] Maggio et al. [7] Twenge et al. [24] Jackson et al. [25]

## Data Availability

Not applicable.
